# Management of Pre-existing Ventriculoperitoneal Shunt in Posterior Vault Distraction for Lambdoid Craniosynostosis: A Case Report and Technical Note

**DOI:** 10.7759/cureus.12814

**Published:** 2021-01-20

**Authors:** Luke H Pearson, Meena Thatikunta, Mohammed Nuru, Scott Rapp, Ian Mutchnick

**Affiliations:** 1 Neurosurgery, University of Louisville Hospital, Louisville, USA; 2 Plastic Surgery, Norton Children's Hospital, Louisville, USA; 3 Neurosurgery, Norton Children's Hospital, Louisville, USA

**Keywords:** chiari i malformation, posterior vault distraction, lambdoid craniosynostosis, vp shunt

## Abstract

Posterior vault distraction osteogenesis (DO) is an emerging treatment option for craniosynostosis. Operative nuances detailing surgical management are being described with increasing use and experience. In this article, we discuss the surgical management of an 8-month-old male with a ventriculoperitoneal shunt (VPS) diagnosed with bilateral lambdoid craniosynostosis and Chiari I malformation. The patient underwent successful bilateral posterior fossa DO without surgical re-implantation of the shunt. Pre- and post-operative imaging confirmed no migration of the VPS. Intracranial volume increased by 20.1% and posterior fossa volume increased by 39.9%. Our experience illustrates that posterior vault DO can be done safely in the setting of a parieto-occipital VPS, in a single operative setting, without the need of additional procedures.

## Introduction

Lambdoid craniosynostosis is rare, and the least common major vault synostoses. It comprises of only 1%-3% of all craniosynostosis cases. The literature on the topic is relatively lacking; it may be overlooked due to its lower proportion of annual cases [1,2]. Bilateral lambdoid synostosis represents an even smaller group of cases. Distinctively, untreated lambdoid synostosis may lead to growth restriction of the posterior fossa and foramen magnum, and depending on the extent of involvement of lambdoid arch sutures, it may cause compression induced neurologic pathology such as tonsillar herniation, hydrocephalus or syrinx [3-7]. Surgical management is the definitive treatment for craniosynostosis, of which, distraction osteogenesis (DO) is an emerging treatment option that is currently employed at 12% of centers nationally. A distinct part of the DO procedure is the relatively rapid skull expansion - occurring at about 1 mm distraction per day for several weeks - implemented by corrective vector forces from an implanted distraction device. For patients with a parieto-occipital ventriculoperitoneal shunt (poVPS), posterior vault DO places the shunt at risk for both infection and migration. Although others have described cases of DO with pre-existing shunt, none have described operative strategies to avoid shunt migration in a rapidly expanding calvarium [8-10]. Here we report surgical details and follow-up results from our experience managing a posterior vault distraction patient with a poVPS in a single operative setting.

## Case presentation

**Figure 1:**
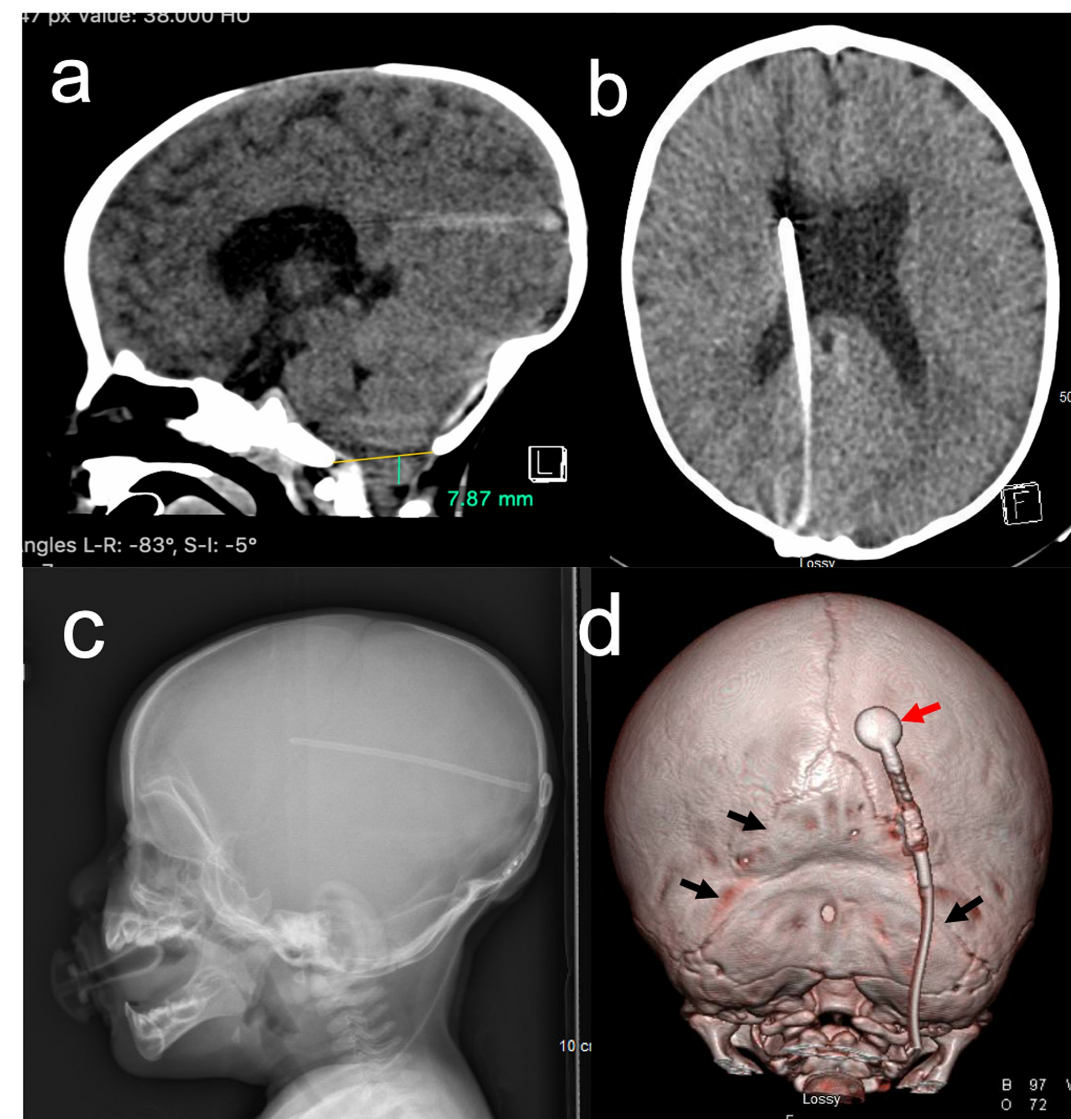
Preoperative CT and x-ray Preoperative axial CT image (a and b), x-ray (c) and 3D modeling (d) demonstrate bilateral lambdoid synostosis with flattening and deformation of the occipital bone and restriction at the level of the foramen magnum. Panel (a) demonstrates tonsillar herniation of 7.87 mm (green) below McRae line (in yellow). Shunt hardware is present at the traditional parieto-occipital entry point with valve (red arrow) and distal tubing overlying the operative area of interest (black arrows) for synostosis surgery.

A 5-month-old male presented to neurosurgery clinic with plagiocephaly and ventriculomegaly. He was born at 36 6/7 weeks by caesarian section. His delivery was complicated by chorioamnionitis, macrosomia, and polyhydramnios. Echocardiogram at one day of age demonstrated tetralogy of Fallot. At birth, head circumference was at the 90th percentile and dropped to 20th percentile at two months of age. No x-ray or CT imaging had been performed at that time. Neurology workup was suggestive of developmental delay and progressive ventriculomegaly. MRI of the head showed mildly dilated lateral and third ventricles without evidence of an obstructing mass or lesion (a communicating cavum septum pellucidum cyst was also noted). MRI also showed a small posterior fossa with mild cerebellar tonsillar ectopia. The etiology of the hydrocephalus was believed to be due to pressure on the sphenoid sinuses from crowding of the small posterior fossa (Figure 1a). Posterior fossa synchondrosis was normal and jugular foramen were both patent. On exam, he was noted to have decreased tone, left esotropia, and nystagmus. Ophthalmologic consultation demonstrated papilledema bilaterally and the patient underwent insertion of a poVPS with a fixed pressure valve (130 mmHg) at five months.

At seven months of age, follow-up cranial CT was ordered to evaluate plagiocephaly. CT demonstrated bilateral lambdoid craniosynostosis (Figure 1d) and mildly increased tonsillar ectopia consistent with Chiari I malformation. The patient’s synostosis was deemed non-syndromic after whole exome sequence testing results were negative.

Although shunt-induced craniosynostosis could not be fully ruled out as the cause of synostosis, we note that the shunt was performed only two months in advance of his craniosynostosis diagnosis. There was no significant drop in the patient’s head circumference, and there were no lambdoid ridges consistent with shunt-induced craniosynostosis. In accordance with our institutional practice, he underwent posterior vault DO with the craniofacial team.

Procedure

Preoperative CT, shown in Figure 1, demonstrates bilateral lambdoid synostosis with rostral portions of the suture still patent bilaterally. The poVPS can also be seen lateral to the sagittal suture with the valve laying over the synostotic mid-section of the right lambdoid.

After intubation and appropriate intravenous access were obtained, the patient was placed in a prone and padded position, and prepped and draped in the sterile fashion. The bicoronal incision site was marked and then infiltrated with 8 ml of 0.5% lidocaine with 1:200,000 epinephrine. An incision was established with a skin knife and bipolar cautery used to gain adequate hemostasis. The shunt was exposed and carefully lateralized. The periosteal plane was disrupted to allow the shunt to remain in place as the skin flap was formed and retracted. Subperiosteal dissection was carried down to 2 cm below the asterion bilaterally. Occipitalis muscle was then removed down to the course of the foramen magnum. Using a highspeed drill, osteotomies were then performed bilaterally.

**Figure 2:**
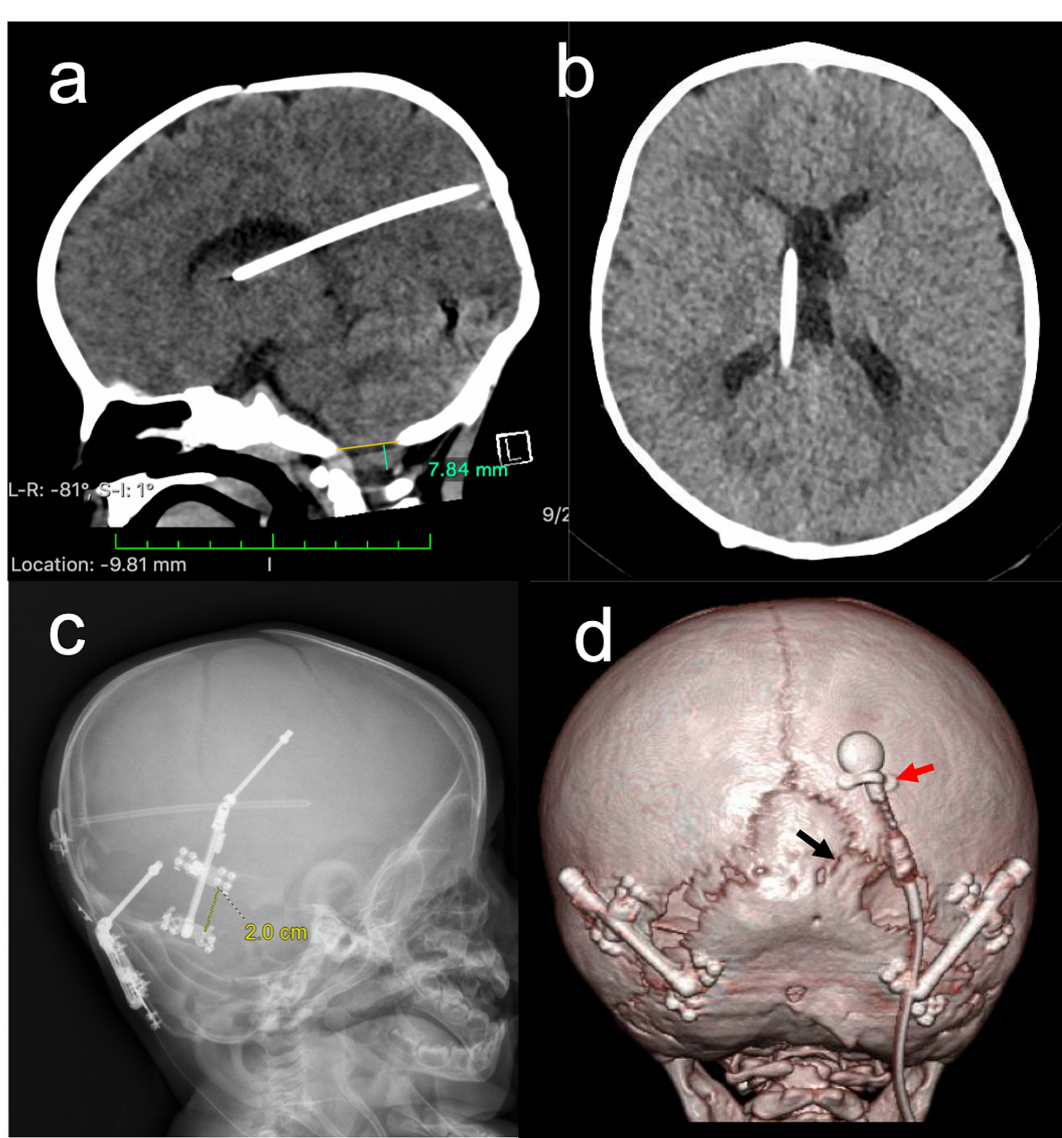
Postoperative CT and x-ray Axial CT image (a and b), x-ray (c) and 3D modeling (d). Panel (c) demonstrates bilateral distractor placement achieving 20 mm of distraction at two weeks' post-op. 3D reconstruction (d) of CT imaging collected at 2.5 months' post-op (a and b) shows evidence of bony bridging across the strip craniectomy (black arrow). Osteotomy was taken to the asterion bilaterally. Shunt is affixed to the skull using a wishbone cranial plate (red arrow). Shunt shows no evidence for migration with distraction.

The osteotomy extended upwards from one asterion along the lambdoid suture, excising it, until the patent rostral section was encountered. From there, the cut ran transversely to meet up with the rostral aspect of the synostosed contralateral lambdoid suture, then heading caudally to the contralateral asterion. In order to remove the section of the right lambdoid underlying the shunt, this hardware was rotated laterally with great care to allow purchase of the drill. While exposed and manipulated, the shunt hardware was meticulously wrapped in a 3.5% betadine-soaked sponge. Once the right suture was cut, the shunt hardware was carefully rotated back into place. Once the lambdoid sutures were excised, neurogen was placed underneath the bone flaps and 30 mm KLS Martin distractors were placed bilaterally. Lastly, a wishbone cranial plate was placed immediately distal to the reservoir (Figure 2d) further fastening the shunt in place. A blood loss of 20 cc was noted and there were no complications.

Distraction and follow-up

**Figure 3:**
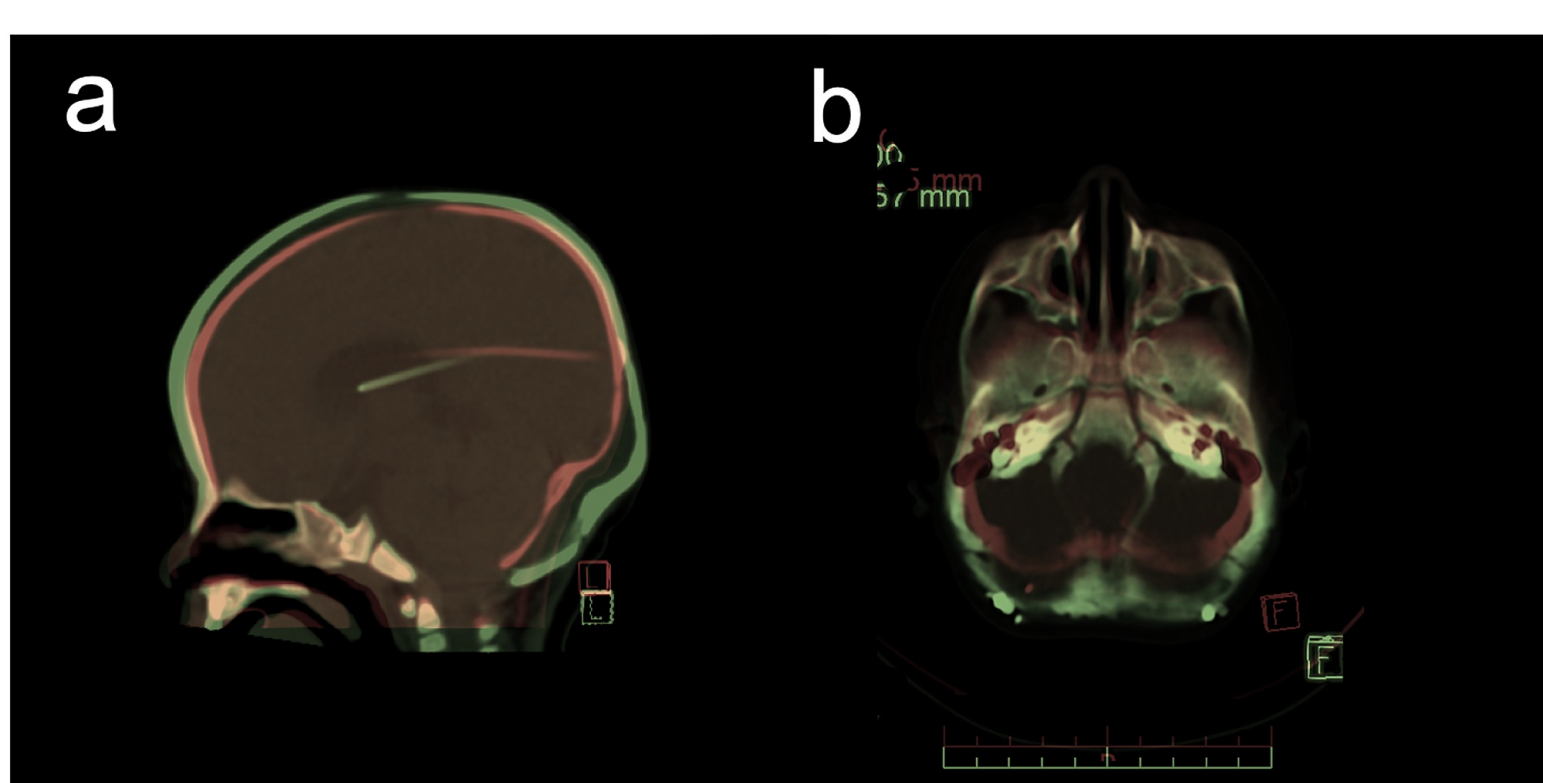
Computerized tomography scan overlay The extent of change to the posterior fossa after distraction can be appreciated in the saggital CT (a) and axial CT overlay (b) shown. Pre-op CT is shown in 'red',  and post-distraction is shown in 'green'.

**Figure 4:**
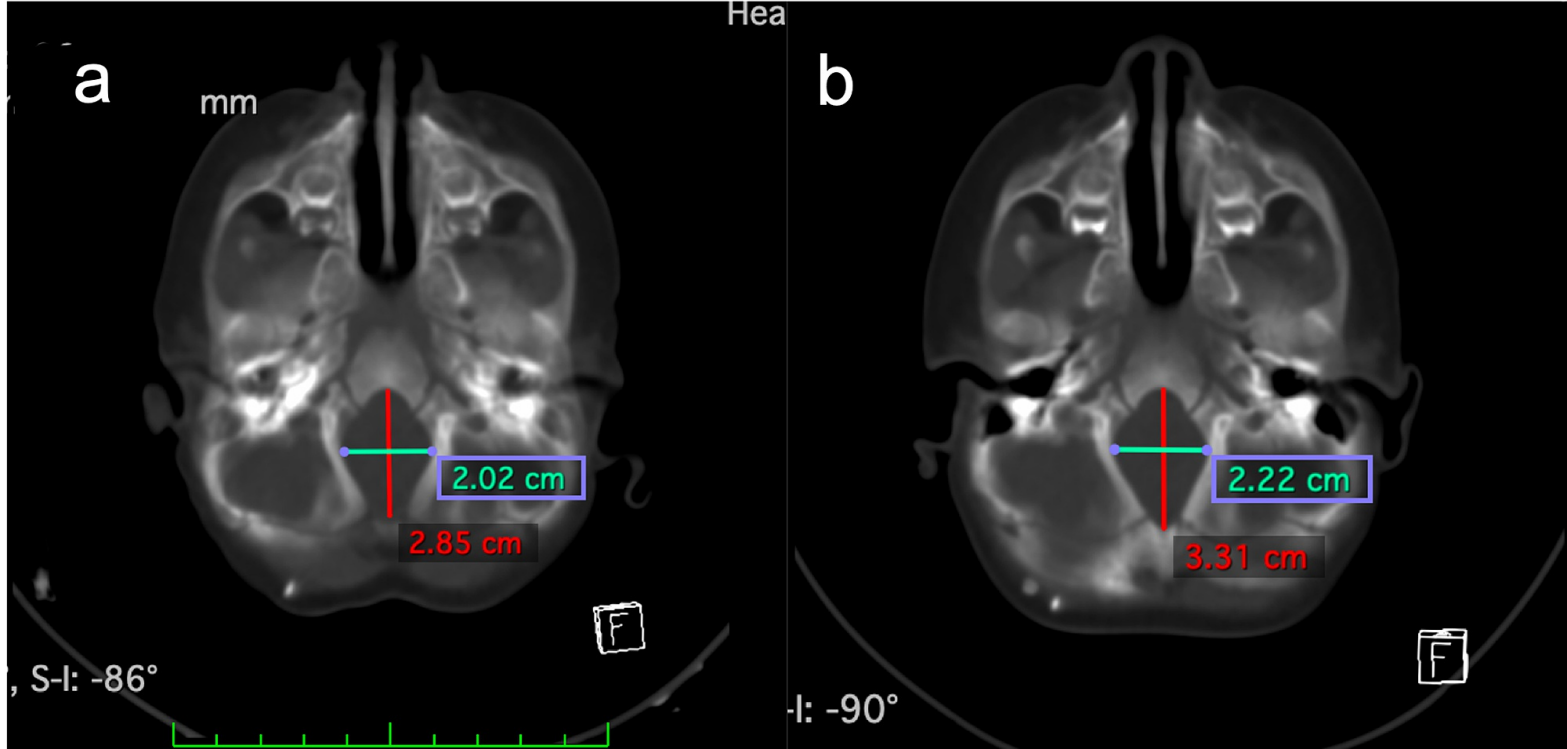
Foramen magnum Pre-operative (a) and post-distraction (b) axial CT dimensions of the foramen magnum.

Distraction was performed at a rate of 1.2 mm per day bilaterally, starting on post-operative day two, for 20 total days of distraction (active phase). Interval skull x-ray performed weekly during distraction demonstrated no migration of the VPS. Total intracranial volume preoperatively measured 993 cc and increased to 1198 cc (21.1% increase) post-operatively. Posterior fossa volume measured 115 cc preoperatively and increased to 161 cc (39.9% increase) post-operatively (Figure 3). When accounting for normal growth of the cranium, the posterior fossa volume was increased by 15.9% by distraction forces. Foramen magnum length and width measured at 2.85 cm and 2.02 cm, respectively, and increased to 3.31 cm (16.1% increase) and 2.22 cm (9.9% increase) (Figure 4). Final distraction distance was 20 mm. The distractor arms were removed at 10 weeks postoperatively.

**Figure 5:**
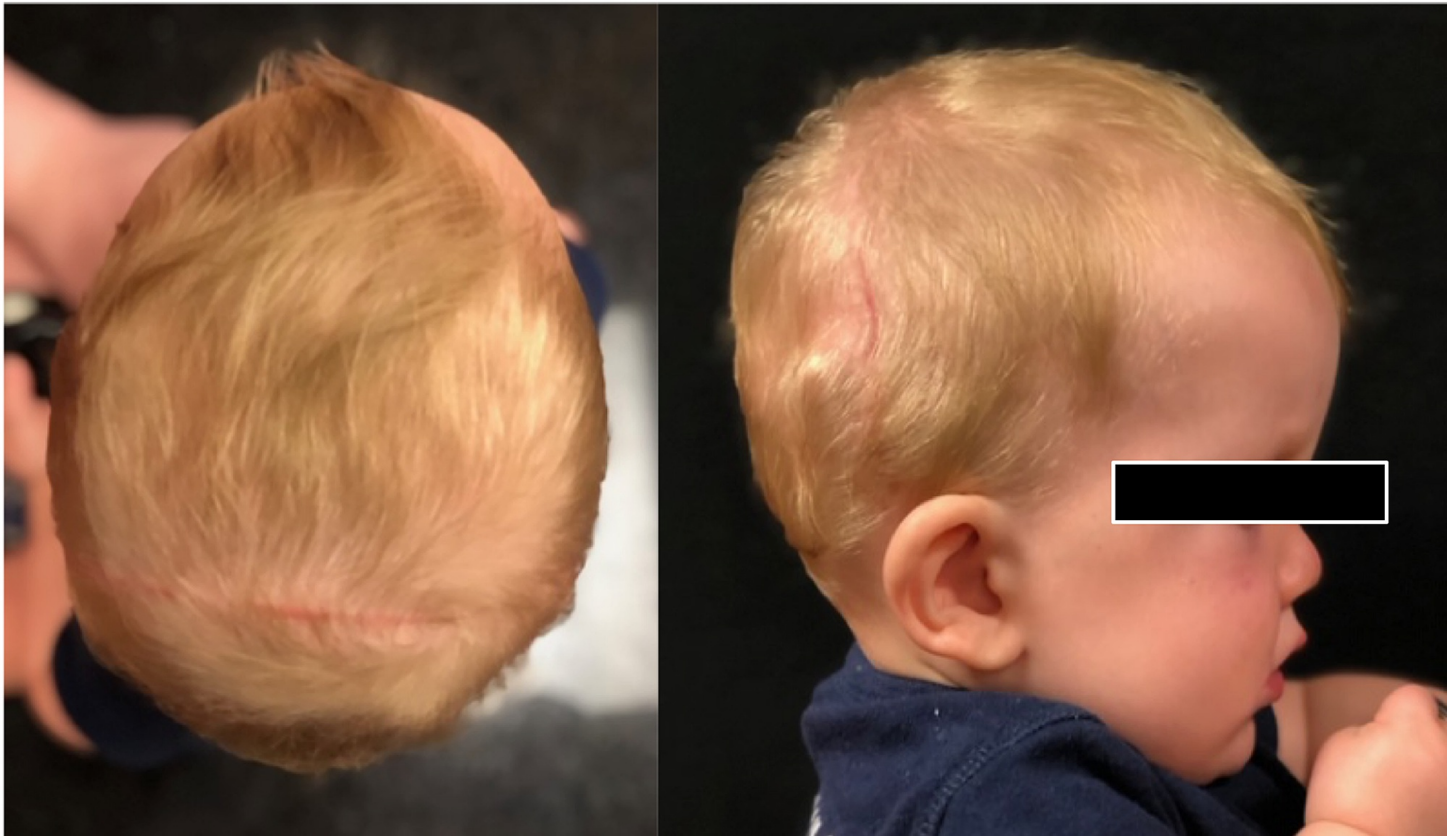
Post-operative cosmetic result Post-operative clinical results at three months demonstrate a good clinical result with improved cranial volume and contour. Incisions are well healed.

CT head obtained 2.5 months postoperatively demonstrated stable VPS placement, without migration, and bony bridging across distracted distance (Figure 2d). Imaging also showed a substantial decrease in the size of the lateral ventricles; with greatest decrease shown in the right lateral horn (Figure 2b). Clinical photos at three-month follow-up showed symmetric and expanded cranial shape (Figure 5). At six-month follow-up, the patient showed progression with developmental milestones and improved radiographic appearance of tonsillar herniation. Head circumference increased to 92nd percentile by six-month post-op, from 51st percentile at the time of surgery. Ventricles were unchanged in size compared to 2.5-month post-op imaging (Figures 2a, 2b).

## Discussion

The development of the posterior fossa is important for the underlying development of the cerebellum, foramen magnum and craniocervical junction. Restricted growth from lambdoid craniosynostosis can lead to a small posterior fossa, which has been associated with the development of hydrocephalus, increased intracranial pressure, and Chiari [1,11]. Treatment options for lambdoid craniosynostosis are limited to endoscopic strip craniectomy (and adjuvant helmet therapy), open cranial vault remodeling and DO. There are advantages and disadvantages to each approach but practically, the selection is limited by surgeon experience. At our institution, we have migrated from open vault reconstruction to DO. This technique involves the release of the stenosed suture and the implantation of a transcutaneously accessible distraction device(s), from which active distraction of the calvarium is performed over a period of time. In this article, we discuss the details of managing a poVPS in a patient undergoing posterior vault DO, emphasizing the learning points for those early in their practice of DO techniques.

A significant advantage of DO is the ability to obtain robust and gradual cranial expansion despite the viscoelastic counterforces of the scalp [5,12]. In contrast, volume expansion in open cranial vault remodeling is more immediate but is limited in extent by these forces at the time of closing. Posterior vault DO can achieve 10% to 34% of steady post-operative increase in total intracranial volume [13-15]. In this patient, we achieved a posterior fossa volume increase of 15.9% from distraction forces alone. The expansion by DO also benefits from soft tissue correction by allowing more gradual stretch and recovery of soft tissue against vector forces of expansion. In this report, our patient showed excellent soft tissue healing over operative site, shunt area, and area of distraction. In this case example, the shunt location did not hinder the ideal placement and orientation of the distractors in the caudal third of the suturectomy near the asterion. Utilizing the most horizontal distraction vector possible in the axial plane, in the lower third of the osteotomy, has demonstrated greater volumetric gains and may have an effect on foramen magnum area and Chiari malformation [16].

In patients with a poVPS, shunt hardware migration from such dynamic expansion is a concern. To avoid shunt migration, we used a wishbone cranial plate immediately distal to the reservoir. This likely helped to protect the shunt, from displacement by vector forces of the cranial expansion process, by adding reinforcement to the shunt’s attachment point on the skull. This was felt to be a critical step, especially after the dissection of surrounding tissues required to execute this particular DO operation. It should also be noted that shunt manipulation during any procedure does increase the risk of shunt malfunction. Regular interval follow-up and x-ray imaging in our DO protocol help to balance against this risk. Interval imaging and family education for early identification of shunt failure are essential.

Bleeding and operative time are other surgical factors to consider in craniosynostosis surgery. DO has been shown to have lower intra-operative bleeding and shorter operative times compared to open vault remodeling [6,17]. Park et al. demonstrate a statistically significant difference in operative time in DO compared to traditional craniectomy (250.9 minutes vs 416.0 minutes, P < 0.001) [6]. This has also been our experience. Our operative time in our published DO cohort was 161± 61 minutes [16]. We have also found that treatment of lambdoid synostosis results in a greater expansion of the foramen magnum. Our morphometric study of 11 lambdoid craniosynostosis patients, produced a significant increase in foramen magnum area with posterior distraction (22% in syndromic, and 26.9% in non-syndromic patients) [16]. The patient described in this report experienced an increase in transverse diameter of 9.9% and an even larger anteroposterior diameter increase of 16.9% resulting in an increased foramen magnum area of 7.85% (not shown). Following this procedure, the patient's Chiari did not progress and remained stable at three- and six-month follow-up. Ventriculomegaly of the lateral ventricles was notably improved on post-operative imaging (Figure 2b).

In patients with a poVPS, the risk of shunt infection from the open procedure and placement of trans-cutaneous hardware for six to eight weeks, as seen in DO, is an added consideration. Reported wound infection rate following DO is 13%, while VPS complication rates range from 3% to 20%. To minimize infection intraoperatively, shunt hardware was protected with a 3.5% betadine solution wrapping. We must stress that there is no substitute for great sterile operative technique and wound care in minimizing the risk of infection. Protecting shunt hardware is an added measure to infection risk minimization. Both physical protection of the shunt during distraction and intraoperative mitigation of infection risk certainly arose as the result of close collaboration between plastic and pediatric neurological surgery.

In this patient case, with bilateral lambdoid craniosynostosis and an existing VPS, we opted to perform DO in a single operative setting. Other options included re-siting the shunt or performing an endoscopic third ventriculostomy (ETV) prior to distraction. These, however, would have required separate (additional) surgeries; and in this patient’s case, his ETV success score (ETVSS) was 40. Giving him a low chance of ETV success by stratified ETVSS (low is ≤ 40; moderate is 50-70; high is ≥80). This said, the addition of a choroid plexus coagulation could indeed increase the chances of ETV success [18].

This experience shows that even in the presence of poVPS, DO can be performed safely, demonstrate a 39.9% increase in posterior fossa volume over a period of three weeks, and lead to neither shunt failure nor infection. At post-operative follow-up at three months and six months, there were no clinical or radiological concerns for shunt migration or infection.

## Conclusions

DO is an emerging method for craniosynostosis treatment,which provides generous volume expansion with direct distraction. As experience with DO increases, operative nuances are receiving more attention in the literature. We describe management of a poVPS in a patient with bilateral lambdoid synostosis undergoing posterior vault DO. Minimizing exposure of shunt hardware, by using a betadine wrapping, and the placement of cranial plate over the shunt valve were sufficient to protect from shunt migration. At six months, the patient’s shunt remains in a stable position and without malfunction or infection, and generous volumetric expansion was achieved.

## References

[1] Rhodes JL, Tye GW, Fearon JA (2014). Craniosynostosis of the lambdoid suture. Semin Plast Surg.

[2] Shillito J, Matson DD (1968). Craniosynostosis: a review of 519 surgical patients. Pediatrics.

[3] Cinalli G, Renier D, Sebag G, Sainte-Rose C, Arnaud E, Pierre-Kahn A (1995). Chronic tonsillar herniation in Crouzon's and Apert's syndromes: the role of premature synostosis of the lambdoid suture. J Neurosurg.

[4] Cinalli G, Sainte-Rose C, Em K (1998). Hydrocephalus and craniosynostosis. J Neurosurg.

[5] Cinalli G, Spennato P, Sainte-Rose C (2005). Chiari malformation in craniosynostosis. Childs Nerv Syst.

[6] Park DH, Chung J, Yoon SH (2010). Rotating distraction osteogenesis in 23 cases of craniosynostosis: comparison with the classical method of craniotomy and remodeling. Pediatr Neurosurg.

[7] Thompson DN, Harkness W, Jones BM, Hayward RD (1997). Aetiology of herniation of the hindbrain in craniosynostosis. An investigation incorporating intracranial pressure monitoring and magnetic resonance imaging. Pediatr Neurosurg.

[8] Bhadkamkar MA, Albright SB, Wolfswinkel EM, Bollo R, Buchanan EP (2015). Posterior cranial vault distraction in the treatment of shunt-induced craniosynostosis. J Craniofac Surg.

[9] de Lima MH, Harshbarger RJ, George TM (2013). Treatment of cephalocranial disproportion in shunt-induced slit ventricle syndrome with cranial vault distraction osteogenesis. Pediatr Neurosurg.

[10] Sandler AL, Daniels LB, Staffenberg DA, Kolatch E, Goodrich JT, Abbott R (2013). Successful treatment of post-shunt craniocerebral disproportion by coupling gradual external cranial vault distraction with continuous intracranial pressure monitoring. J Neurosurg Pediatr.

[11] Rijken BF, Lequin MH, Van der Lijn F (2015). The role of the posterior fossa in developing Chiari I malformation in children with craniosynostosis syndromes. J Craniomaxillofac Surg.

[12] Ter Maaten NS, Mazzaferro DM, Wes AM, Naran S, Bartlett SP, Taylor JA (2018). Craniometric analysis of frontal cranial morphology following posterior vault distraction. J Craniofac Surg.

[13] Salokorpi N, Vuollo V, Sinikumpu JJ (2017). Increases in cranial volume with posterior cranial vault distraction in 31 consecutive cases. Neurosurgery.

[14] Serlo WS, Ylikontiola LP, Lahdesluoma N (2011). Posterior cranial vault distraction osteogenesis in craniosynostosis: estimated increases in intracranial volume. Childs Nerv Syst.

[15] Shimizu A, Komuro Y, Shimoji K, Miyajima M, Arai H (2016). Quantitative analysis of change in intracranial volume after posterior cranial vault distraction. J Craniofac Surg.

[16] Thatikunta M, Pearson L, Nguyen C (2020). Three-dimensional volumetric changes in posterior vault distraction with distraction osteogenesis. J Craniofac Surg.

[17] Akizuki T, Komuro Y, Ohmori K (2000). Distraction osteogenesis for craniosynostosis. Neurosurg Focus.

[18] Riva-Cambrin J, Kestle JRW, Rozzelle CJ (2019). Predictors of success for combined endoscopic third ventriculostomy and choroid plexus cauterization in a North American setting: a Hydrocephalus Clinical Research Network study. J Neurosurg Pediatr.

